# Clinical-based phenotypes in children with pediatric post-COVID-19 condition

**DOI:** 10.1007/s12519-024-00805-2

**Published:** 2024-04-25

**Authors:** Lieke C. E. Noij, Jelle M. Blankestijn, Coen R. Lap, Marlies A. van Houten, Giske Biesbroek, Anke-Hilse Maitland-van der Zee, Mahmoud I. Abdel-Aziz, Johannes B. van Goudoever, Mattijs W. Alsem, Caroline L. H. Brackel, Kim J. Oostrom, Simone Hashimoto, Suzanne W. J. Terheggen-Lagro

**Affiliations:** 1 Department of Pediatric Pulmonology and Allergy, Amsterdam UMC, Location AMC, Emma Children’s Hospital, Amsterdam University Medical Center, Room G2-222, Meibergdreef 9, 1105AZ Amsterdam, The Netherlands; 2 Department of Pulmonary Medicine, Amsterdam UMC, Amsterdam, The Netherlands; 3 Department of Pediatrics, Spaarne Gasthuis, Hoofddorp, The Netherlands; 4 Department of Pediatrics, Emma Children’s Hospital, Amsterdam University Medical Center, Amsterdam, The Netherlands; 5 Department of Rehabilitation Medicine, Amsterdam Movement Sciences, Meibergdreef 9, Amsterdam, The Netherlands; 6 Department of Paediatric Pulmonology, Department of Paediatrics, Tergooi MC, Hilversum, The Netherlands; 7 Department of Child and Adolescent Psychiatry and Psychosocial Care, Amsterdam UMC, Amsterdam Reproduction and Development, Amsterdam, The Netherlands

**Keywords:** Adolescents, Clusters, Long COVID, Pediatric, Phenotypes, Post-COVID syndrome

## Abstract

**Background:**

Pediatric post coronavirus disease 2019 (COVID-19) condition (PPCC) is a heterogeneous syndrome, which can significantly affect the daily lives of children. This study aimed to identify clinically meaningful phenotypes in children with PPCC, to better characterize and treat this condition.

**Methods:**

Participants were children with physician-diagnosed PPCC, referred to the academic hospital Amsterdam UMC in the Netherlands between November 2021 and March 2023. Demographic factors and information on post-COVID symptoms, comorbidities, and impact on daily life were collected. Clinical clusters were identified using an unsupervised and unbiased approach for mixed data types.

**Results:**

Analysis of 111 patients (aged 3–18 years) revealed three distinct clusters within PPCC. Cluster 1 (*n* = 62, median age = 15 years) predominantly consisted of girls (74.2%). These patients suffered relatively more from exercise intolerance, dyspnea, and smell disorders. Cluster 2 (*n* = 33, median age = 13 years) contained patients with an even gender distribution (51.5% girls). They suffered from relatively more sleep problems, memory loss, gastrointestinal symptoms, and arthralgia. Cluster 3 (*n* = 16, median age = 11 years) had a higher proportion of boys (75.0%), suffered relatively more from fever, had significantly fewer symptoms (median of 5 symptoms compared to 8 and 10 for clusters 1 and 2 respectively), and experienced a lower impact on daily life.

**Conclusions:**

This study identified three distinct clinical PPCC phenotypes, with variations in sex, age, symptom patterns, and impact on daily life. These findings highlight the need for further research to understand the potentially diverse underlying mechanisms contributing to post-COVID symptoms in children.

**Graphical abstract:**

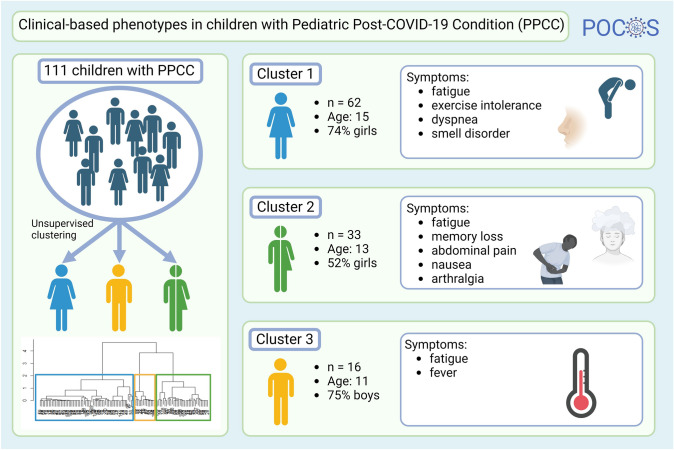

**Supplementary Information:**

The online version contains supplementary material available at 10.1007/s12519-024-00805-2.

## Introduction

In March 2020, the World Health Organization (WHO) declared the coronavirus disease 2019 (COVID-19) outbreak a global pandemic [1], affecting millions of people worldwide. COVID-19 is caused by severe acute respiratory syndrome coronavirus-2 (SARS-CoV-2), and while the initial focus was on pulmonary symptoms, it is now well established that COVID-19 can have diverse disease presentations and involve multiple organ systems [2]. Although children are less likely to experience severe disease than adults, they can still be affected by COVID-19 and develop long-term symptoms [3–5]. The persistence of symptoms for longer than 12 weeks after COVID-19 is known as post-COVID-19 condition [6]. This condition in children will henceforth be referred to as pediatric post-COVID-19 condition (PPCC).

The reported occurrence of PPCC varies widely among studies, with a large systematic review [7] from May 2023 reporting a pooled prevalence of 23%, with prevalence rates ranging from 3.7% to 66% [7–9]. This wide range could be caused by differences in study populations (e.g., hospitalized versus. non-hospitalized children), different follow-up approaches, or lack of control groups [10]. PPCC is a heterogeneous illness, characterized by a broad range of symptoms and the involvement of multiple organ systems. The most common complaints are fatigue, mood changes, headache, cognitive difficulties (“brain fog”), dyspnea, and loss of smell [4, 10]. Other symptoms related to PPCC include tachycardia, chest pain, cough, abdominal pain, nausea and lack of appetite, (recurrent) fever, post-exertional malaise, sleep dysfunction, dizziness, skin rashes, and joint and muscle pains [11]. Risk factors for long-term symptoms after COVID-19 in children that have previously been described are older age (> 10 years), female gender, comorbidities (e.g., allergies, neurologic or genetic diseases), and hospitalization during the acute phase [12, 13].

PPCC can have a profound effect on daily life [12], necessitating a growing societal demand for diagnostic and treatment strategies. This has led to the emergence of PPCC clinics all over the world and an international knowledge exchange platform, consisting of physicians, allied-health professionals, and patient representatives [14]. However, the treatment strategies employed vary greatly among these clinics and are mostly based on experience. This is partly associated with a lack of understanding of the pathophysiological mechanisms underlying PPCC. Recent research hypothesizes that post-COVID-19 condition in adults may be caused by immune dysregulation (e.g., due to viral persistence), microbiota disruption, autoimmunity, clotting and endothelial abnormality, or dysfunctional neurologic signaling [15]. However, it is unclear if similar mechanisms are responsible for post-COVID-19 condition in children.

From clinical practice, we observed a diversity of symptoms associated with PPCC [14], yet certain characteristics seem to cluster together. Buonsenso et al. [16] described symptom patterns, grouping them (supervised clustering) based on organ systems. Unsupervised clustering of symptoms in adults with post-COVID-19 condition on separate occasions has shown that such a group can be clustered into phenotypes with different organ system involvement and severity [17, 18]. However, this has not yet been performed in children with PPCC. Unsupervised clustering of PPCC symptoms and characteristics could provide inherent patterns that may represent distinct phenotypes.

The aim of this study was to identify clinically meaningful phenotypes of PPCC. Understanding the clinical features of PPCC may help us to better characterize this condition, thereby expanding our knowledge of potential underlying pathophysiological mechanisms.

## Methods

### Study design

This study used data collected in the post-COVID syndrome (POCOS) study, a prospective, observational cohort study conducted at the Amsterdam University Medical Center (Amsterdam UMC), a tertiary care hospital based in Amsterdam, the Netherlands. The POCOS study investigates PPCC in the pediatric population, aiming to describe clinical characteristics, investigate underlying mechanisms, and identify biomarkers for PPCC. Data from patients was collected from medical files and during patient visits. The medical ethics committee of the Amsterdam UMC, location AMC, approved the study (METc 2021_126). All participants and/or caregivers provided oral and written informed consent.

### Participants and procedures

From November 2021 to March 2023, Dutch children (aged 0–18 years) with PPCC referred to the post-COVID multidisciplinary outpatient clinic of the Amsterdam UMC were consecutively invited to participate in the POCOS study. The inclusion criteria were: (1) PCC, as diagnosed by a physician according to the WHO definition [6], with a history of at least one positive real-time reverse transcription-polymerase chain reaction (RT-PCR) test on nasopharyngeal, oropharyngeal, sputum, or fecal sample for SARS-CoV-2, or proof of infection with SARS-CoV-2 by positive serology test (immunoglobulin G (IgG)/immunoglobulin M (IgM)), or medical history suitable with acute SARS-CoV-2 infection, if the acute episode occurred between February to June 2020 (due to restricted testing possibilities for children in the Netherlands), and (2) complaints lasting > 12 weeks after acute COVID-19.

### Data collection

#### Demographic and clinical characteristics

Demographic factors, including information on age, sex, level of education, and clinical characteristics such as medical history, comorbidities, symptoms in the acute COVID-19 phase, post-COVID symptoms, and body mass index (BMI) plus Z-scores [19] were collected from all participants, either by outpatient visits with a physician or retrieved from medical files. The suspected SARS-CoV-2 variant was determined by the dominant virus type in the Netherlands at the time of infection based on numbers from the National Institute for Public Health and the Environment (RIVM) [20]. Limitations in daily life were scored for three domains: first, school limitations, with impact scores of 0 (no impact), 1 (mild impact; 1–2 days of absence per month), 2 (moderate impact; 1–2 days of absence per week), and 3 (severe impact; 3–5 days of absence per week). Second, social limitations, with impact scores of 0 (no impact), 1 (mild impact; 1–2 days per month not capable), 2 (moderate impact; 1–2 days per week not capable), and 3 (severe impact; no social contact possible). Third, physical limitations, with impact scores of 0 (no impact), 1 (mild impact; 80% of physical functioning), 2 (moderate impact; 50% of physical functioning), and 3 (severe impact; no physical functioning).

#### Patient-reported outcomes measurement information system (PROMIS) pediatric fatigue questionnaire

Fatigue is the most common symptom of post-COVID-19 condition [5]. PROMIS® Pediatric Fatigue Scale is a questionnaire that uses the Computerized Adaptive Test (CAT) format, in which subsequent questions are chosen based on the answers given earlier. PROMIS Fatigue scores are reported on a T-score metric ranging from 0 to 100, with higher values representing more fatigue [21]. The PROMIS Fatigue has been validated among Dutch children (between 8 and 18 years of age), who showed an average T-score of 39.8 ± 12.4 [22]. For this reason, only children aged ≥ 8 years old received questionnaires. The minimal clinically important difference (MCID) of the PROMIS Fatigue is 2–6 points [23].

### Statistical analyses

#### Data storage

All collected data was stored in an online case report form (CRF) by Castor Electronic Data Capture (EDC) [24], a platform intended for capturing medical research data in clinical trials. The study was closely monitored and the quality of the data was controlled.

#### Imputation

An overview of the variables used for clustering is found in Supplementary Table 1. Variables were only eligible for selection if at most 15% were missing. Missing data was imputed using the Multiple Imputation by Chained Equations (MICE) algorithm as implemented by the mice R package (version 3.15.0) [25]. Imputation for numerical variables was performed using predictive mean matching, binary categorical variables were imputed with logistic regression, ordered categorical variables were imputed with a proportional odds model, and unordered variables were imputed through polytomous logistic regression. One hundred different imputed datasets were created to account for the uncertainty of missing data.

#### Clustering algorithm

The individual datasets were converted to pairwise Gower distance between the patients. Hierarchical clustering based on the Ward.D2 construction method was used to create dendrograms. Visual inspection of the dendrograms and the Dunn index [26] determined the optimal number of clusters that maximize the distances between clusters while minimizing the distances within clusters. To obtain a consensus clustering, pairwise distances from the cluster assignments in individual datasets were calculated for each pair of patients, which was subsequently clustered through hierarchical clustering.

#### Cluster interpretation

Patient demographics and clinical characteristics were compared between the clusters using statistical tests based on the type of data. Categorical variables were compared using Fisher’s exact test, and numerical variables were compared with the Kruskal–Wallis test. Post-hoc testing of the statistical significance of comparisons was performed with pairwise Fisher’s exact tests for categorical variables and pairwise Wilcoxon rank sum tests with correction for multiple testing for numerical variables. Symptom presence or absence between the two largest clusters was compared with an odds ratio as calculated by the epitools package (version 0.5–10.1) [27]. All analyses were performed in R (version 4.1.2) with RStudio (version 2021.09.1 + 372) [28].

## Results

### Cohort demographics

**Fig. 1:**
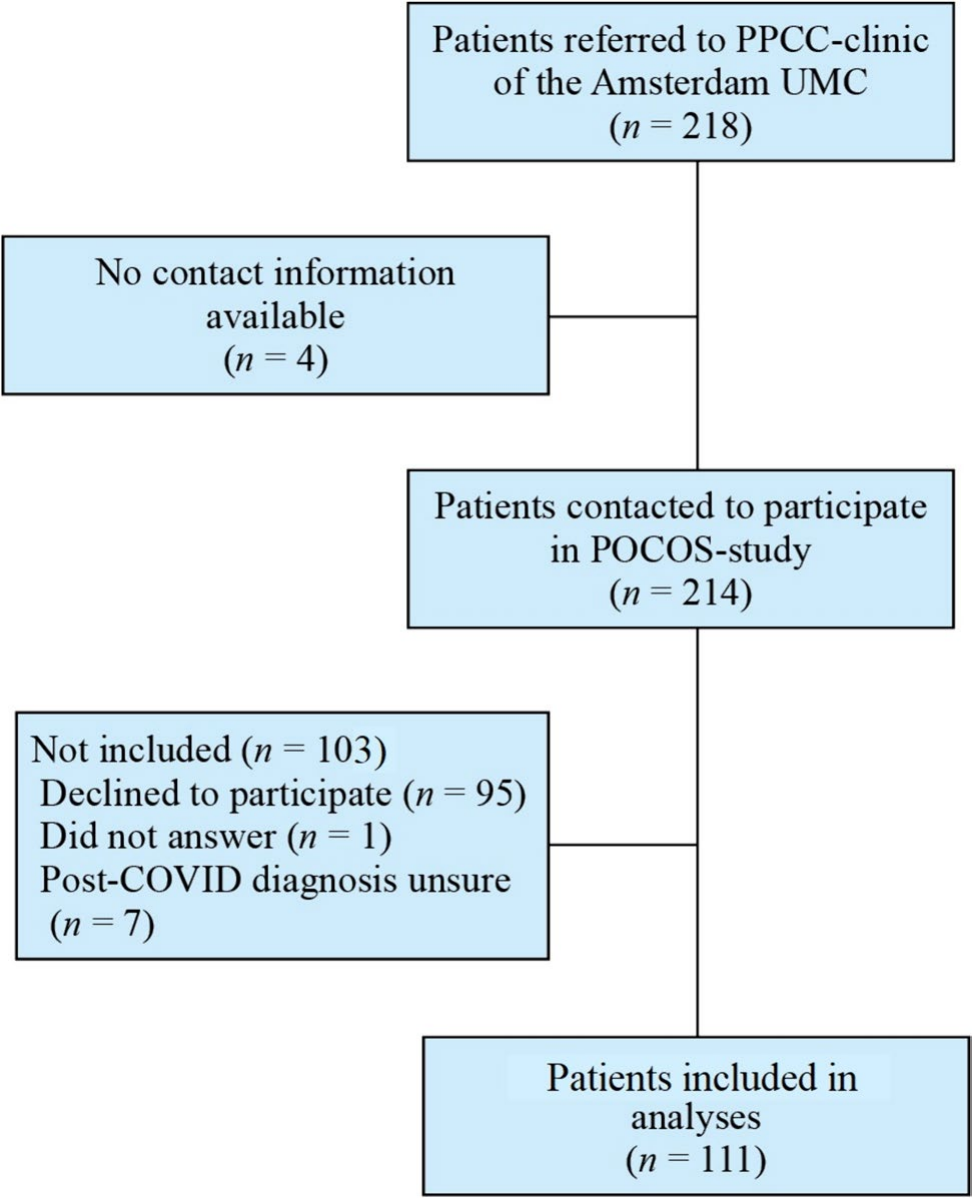
Flow diagram of patients participating in this study between November 2021 and March 2023

In total, 111 participants were included in this study (Fig. 1). The demographic and clinical characteristics of these patients can be found in Table 1. Missing data constituted 1.9% of all data used for clustering. Participants were between 3 and 18 years old, with a median age of 14.0 years [interquartile range (IQR): 11.0–16.0]. The cohort consisted of slightly more girls than boys (60.4% and 39.6% respectively). The most common complaints were fatigue (100.0%), exercise intolerance (80.2%), and oversensitivity/overstimulation (76.6%). Over two-thirds (68.5%) of the participants reported comorbidities. The BMI was normal for children of these ages, with a median z-score of − 0.1 (IQR − 0.7–0.9). All participants had mild acute COVID-19; none were admitted to the hospital during the acute phase.

**Table 1:** Demographic and clinical characteristics of participants, showing all patients and patients separated in three clusters

Variables	Total (*n* = 111)	Cluster 1 (*n* = 62)	Cluster 2 (*n* = 33)	Cluster 3 (*n* = 16)	*P* value
General characteristics					
Sex (male)	44 (39.6%)	16 (25.8%)	16 (48.5%)	12 (75.0%)	** < 0.001**
Age in years^a^	14.0 (11.0, 16.0)	15.0 (13.0, 16.0)	13.0 (9.0, 16.0)	11.0 (8.8, 14.0)	** < 0.001**
BMI (z-score)^a^	− 0.1 (− 0.7, 0.9) (*n *= 103)	− 0.2 (− 0.7, 0.6) (*n* = 58)	* –*0.0 (− 0.4, 1.4) (*n* = 33)	− 0.6 (− 1.3, 0.7) (*n *= 12)	0.055
Family member with post-COVID complaints	51 (54.3%) (*n* = 94)	32 (59.3%) (*n* = 54)	15 (53.6%) (*n* = 28)	4 (33.3%) (*n* = 12)	0.267
Days between infection and study visit^a^	471 (310, 648) (*n* = 95)	488 (342, 646) (*n*=53)	451 (296, 650) (*n* = 30)	406 (262, 628) (*n* = 12)	0.490
Comorbidities^b^					
Asthma	13 (11.7%)	7 (11.3%)	6 (18.2%)	0 (0.0%)	0.202
Allergies	54 (48.6%)	31 (50.0%)	20 (60.6%)	3 (18.8%)	**0.020**
Psychiatric disorders	23 (20.7%)	16 (25.8%)	2 (6.1%)	5 (31.2%)	**0.027**
Other	21 (18.9%)	15 (24.2%)	5 (15.2%)	1 (6.2%)	0.236
None	35 (31.5%)	19 (30.6%)	8 (24.2%)	8 (50.0%)	0.201
Number of SARS-CoV-2 infections	(*n* = *100*)	(*n* = *58*)	(*n* = *28*)	(*n* = *14*)	0.606
One	67 (67.0%)	38 (65.5%)	18 (64.3%)	11 (78.6%)	
Two	30 (30.0%)	19 (32.8%)	8 (28.6%)	3 (21.4%)	
Three	3 (3.0%)	1 (1.7%)	2 (7.1%)	0 (0.0%)	
Dominant virus type^c^	(*n* = *110*)	(*n* = *62*)	(*n* = *32*)	(*n* = *16*)	0.413
Alpha	73 (66.4%)	45 (72.6%)	18 (56.2%)	10 (62.5%)	
Delta	23 (20.9%)	10 (16.1%)	10 (31.2%)	3 (18.8%)	
Omicron	14 (12.7%)	7 (11.3%)	4 (12.5%)	3 (18.8%)	
Acute COVID-19 clinical presentation^d^					
Upper airway infection	86 (77.5%)	50 (80.6%)	24 (72.7%)	12 (75.0%)	0.631
Gastroenteritis	7 (6.3%)	2 (3.2%)	5 (15.2%)	0 (0.0%)	0.056
Other	7 (6.3%)	4 (6.5%)	0 (0.0%)	3 (18.8%)	**0.043**
Asymptomatic	11 (9.9%)	6 (9.7%)	4 (12.1%)	1 (6.2%)	0.909
Vaccination status	(*n* = *108*)	(*n* = *60*)	(*n* = *33*)	(*n* = *15*)	0.543
Not vaccinated	36 (33.3%)	17 (28.3%)	14 (42.4%)	5 (33.3%)	
Vaccinated	70 (64.8%)	42 (70.0%)	18 (54.5%)	10 (66.7%)	
Vaccination timing	(*n* = *69*)	(*n* = *42*)	(*n* = *17*)	(*n* = *10*)	0.132
Vaccinated before infection	13 (18.8%)	5 (11.9%)	5 (29.4%)	3 (30.0%)	
Vaccinated after infection	56 (81.2%)	37 (88.1%)	12 (70.6%)	7 (70.0%)	
Level of education	(*n* = *108*)	(*n* = *61*)	(*n* = *31*)	(*n* = *16*)	**0.026**
Elementary school	33 (30.6%)	11 (18.0%)	13 (41.9%)	9 (56.2%)	
Pre-vocational secondary education	13 (12.0%)	7 (11.5%)	3 (9.7%)	3 (18.8%)	
Senior general secondary education	23 (21.3%)	16 (26.2%)	6 (19.4%)	1 (6.2%)	
Pre-university education	39 (36.1%)	27 (44.3%)	9 (29.0%)	3 (18.8%)	
Impact on daily life—school^e^	(*n* = *105*)	(*n* = *59*)	(*n* = *30*)	(*n* = *16*)	**0.006**
No impact	16 (15.2%)	10 (16.9%)	1 (3.3%)	5 (31.2%)	
Mild impact	23 (21.9%)	11 (18.6%)	5 (16.7%)	7 (43.8%)	
Moderate impact	38 (36.2%)	20 (33.9%)	14 (46.7%)	4 (25.0%)	
Severe impact	28 (26.7%)	18 (30.5%)	10 (33.3%)	0 (0.0%)	
Impact on daily life—social interactions^e^	(*n* = *106*)	(*n* = *60*)	(*n* = *30*)	(*n* = *16*)	0.429
No impact	24 (22.6%)	14 (23.3%)	6 (20.0%)	4 (25.0%)	
Mild impact	30 (28.3%)	14 (23.3%)	9 (30.0%)	7 (43.8%)	
Moderate impact	45 (42.5%)	29 (48.3%)	11 (36.7%)	5 (31.2%)	
Severe impact	7 (6.6%)	3 (5.0%)	4 (13.3%)	0 (0.0%)	
Impact on daily life—physical functioning^e^	(*n* = *107*)	(*n* = *59*)	(*n* = *32*)	(*n* = *16*)	** < 0.001**
No impact	7 (6.5%)	1 (1.7%)	1 (3.1%)	5 (31.2%)	
Mild impact	30 (28.0%)	14 (23.7%)	8 (25.0%)	8 (50.0%)	
Moderate impact	48 (44.9%)	29 (49.2%)	16 (50.0%)	3 (18.8%)	
Severe impact	22 (20.6%)	15 (25.4%)	7 (21.9%)	0 (0.0%)	
PROMIS fatigue	(*n* = *79*)	(*n* = *48*)	(*n* = *20*)	(*n* = *11*)	
Score	62.8 ± 10.1	63.7 ± 9.4	63.7 ± 12.0	57.0 ± 8.2	0.077
Highly prevalent symptoms					
Fatigue	111 (100.0%)	62 (100.0%)	33 (100.0%)	16 (100.0%)	1.000
Exercise intolerance	89 (80.2%)	59 (95.2%)	26 (78.8%)	4 (25.0%)	** < 0.001**
Dyspnea	57 (51.4%)	42 (67.7%)	15 (45.5%)	0 (0.0%)	** < 0.001**
Sleep problems	47 (42.3%)	16 (25.8%)	29 (87.9%)	2 (12.5%)	** < 0.001**
Heart palpitations	34 (30.6%)	20 (32.3%)	12 (36.4%)	2 (12.5%)	0.233
Neurologic symptoms					
Oversensitivity/overstimulation	85 (76.6%)	50 (80.6%)	29 (87.9%)	6 (37.5%)	** < 0.001**
Headache	73 (65.8%)	41 (66.1%)	26 (78.8%)	6 (37.5%)	**0.020**
Memory loss	63 (56.8%)	35 (56.5%)	26 (78.8%)	2 (12.5%)	** < 0.001**
Dizziness	38 (34.2%)	25 (40.3%)	10 (30.3%)	3 (18.8%)	0.230
Concentration loss	37 (33.3%)	26 (41.9%)	9 (27.3%)	2 (12.5%)	0.055
Taste disorders	25 (22.5%)	17 (27.4%)	5 (15.2%)	3 (18.8%)	0.433
Smell disorders	26 (23.4%)	20 (32.3%)	4 (12.1%)	2 (12.5%)	0.062
Gastrointestinal symptoms					
Abdominal pain	34 (30.6%)	7 (11.3%)	20 (60.6%)	7 (43.8%)	** < 0.001**
Nausea	40 (36.0%)	12 (19.4%)	24 (72.7%)	4 (25.0%)	** < 0.001**
Loss of appetite	38 (34.2%)	16 (25.8%)	20 (60.6%)	2 (12.5%)	** < 0.001**
Musculoskeletal symptoms					
Arthralgia	22 (19.8%)	8 (12.9%)	11 (33.3%)	3 (18.8%)	0.067
Myalgia	27 (24.3%)	12 (19.4%)	12 (36.4%)	3 (18.8%)	0.194
Other symptoms					
Fever	16 (14.4%)	5 (8.1%)	6 (18.2%)	5 (31.2%)	**0.042**
Sore throat	12 (10.8%)	6 (9.7%)	5 (15.2%)	1 (6.2%)	0.685
Skin rash	20 (18.0%)	12 (19.4%)	4 (12.1%)	4 (25.0%)	0.543
Hair loss	9 (8.1%)	4 (6.5%)	5 (15.2%)	0 (0.0%)	0.192
Menstrual problems	11/57 (19.3%)	9/42 (21.4%)	1/13 (7.7%)	1/2 (50.0%)	0.237

Bold values represent statistically significant (*p* < 0.05) differences between the three clusters

^a^Median + IQR

^b^Allergy: allergic rhinitis, eczema, food allergy, other allergies. Psychiatric disorder: autism spectrum disorder, attention deficit hyperactivity disorder, other psychiatric disorders. Other: Pfeiffer, migraine, high susceptibility for infections

^c^Dominant SARS-CoV-2-type was determined by the dominant variant in the Netherlands at the time of infection

^d^All patients had a mild acute COVID-19. No patients were admitted to the hospital. “Other” includes: fatigue, pneumonia, asthma exacerbation, smell and taste disorders, mild multi-system inflammatory symptoms

^e^See section *Demographic and clinical characteristics* for clarification of impact

*P* values were determined through Kruskal–Wallis tests for numerical variables and Fisher’s exact tests for categorical variables, and represents if there is a difference in at least one cluster compared to the others

*BMI* Body mass index, *IQR* interquartile range, *PROMIS* The Patient-Reported Outcomes Measurement Information System

#### Differences in patient demographics and comorbidities

**Fig. 2:**
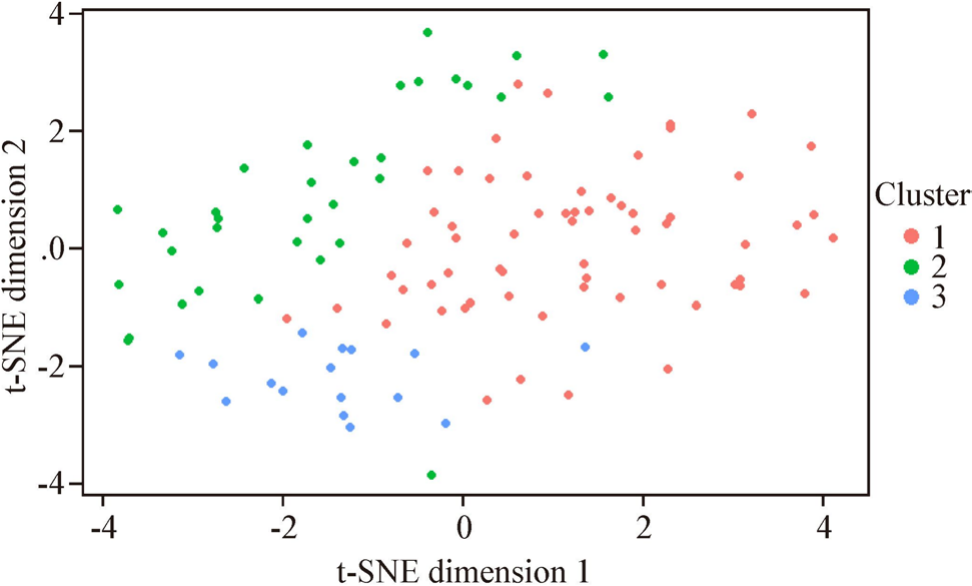
Visualization of the cluster separations using a t-SNE plot of dimension reduction. Data from this plot has not been imputed

Clusters were formed through hierarchical clustering. Based on visual inspection of the dendrograms and the Dunn indices from the clustering of individual datasets, and from the consensus clustering (Supplementary Fig. 1), three clusters were chosen to best represent the data. Cluster separation based on a t-SNE plot can be seen in Fig. 2. In total, 62 patients were placed in cluster 1, 33 patients in cluster 2, and 16 patients in cluster 3. A comparison between the clusters can be found in Table 1. The clusters showed differences in age and sex distribution (*P* < 0.001). Cluster 1 predominantly consisted of girls (74.2%), cluster 2 was more evenly distributed (48.5% boys), while cluster 3 predominantly consisted of boys (75.0%); however, this was not statistically different from cluster 2 or from 50% (*P* = 0.077). Cluster 1 also contained relatively older patients, with a median age of 15.0 years (IQR: 13.0–16.0), compared to a median age of 13.0 years (IQR: 9.0–16.0) in cluster 2 and 11.0 years (IQR: 8.8–14.0) in cluster 3. In terms of comorbidities, there were no differences between the clusters for asthma, however, cluster 3 contained significantly fewer patients with allergies (18.8% compared to 50.0% and 60.6% in cluster 1 and 2, respectively) and cluster 2 contained significantly fewer patients with psychiatric disorders (6.1% compared to 25.8% and 31.2% in cluster 1 and 3, respectively).

#### Differences in patterns of symptoms

**Fig. 3:**
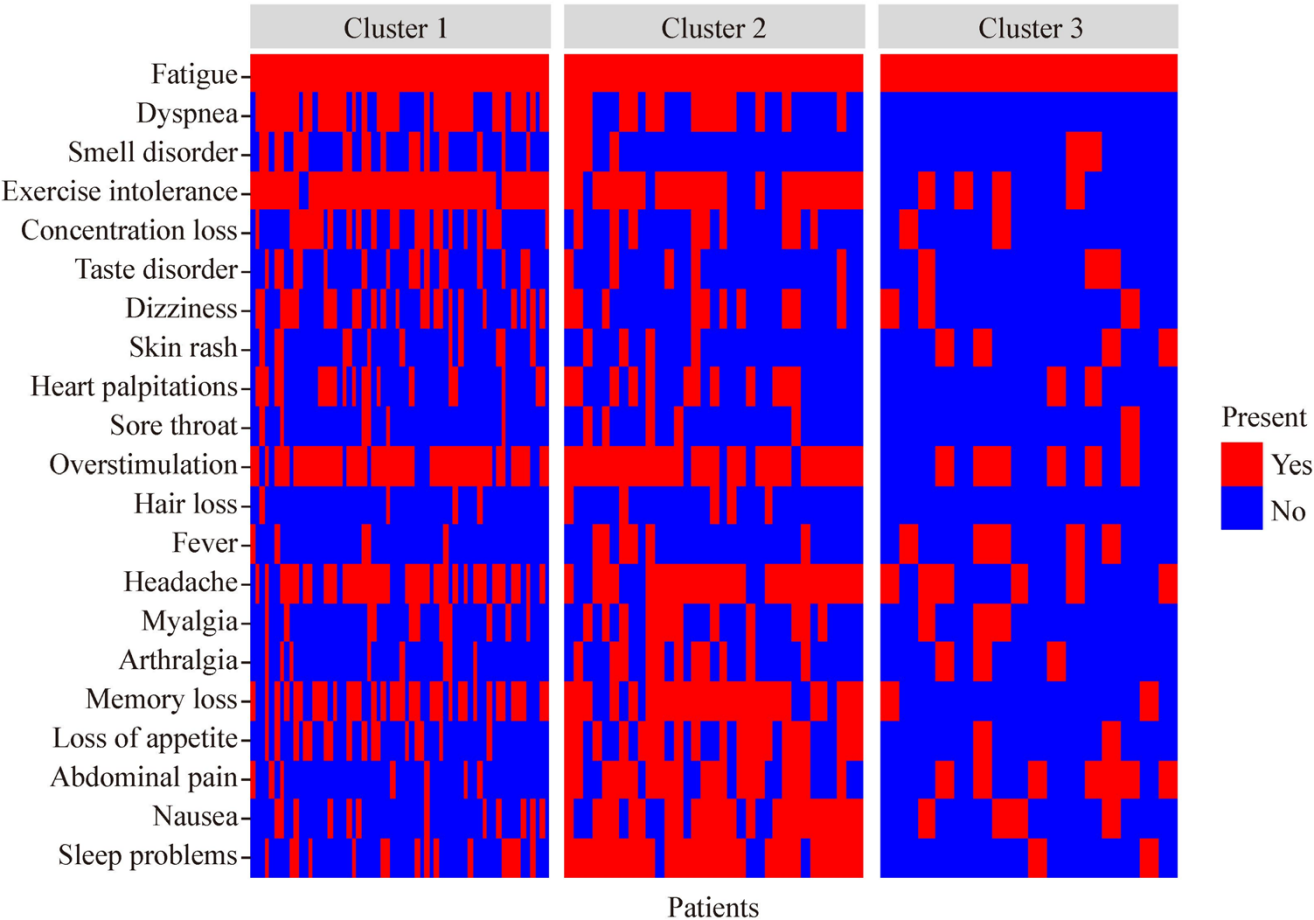
Heat map depicting the presence of absence of symptoms of the patients separated by cluster

**Fig. 4:**
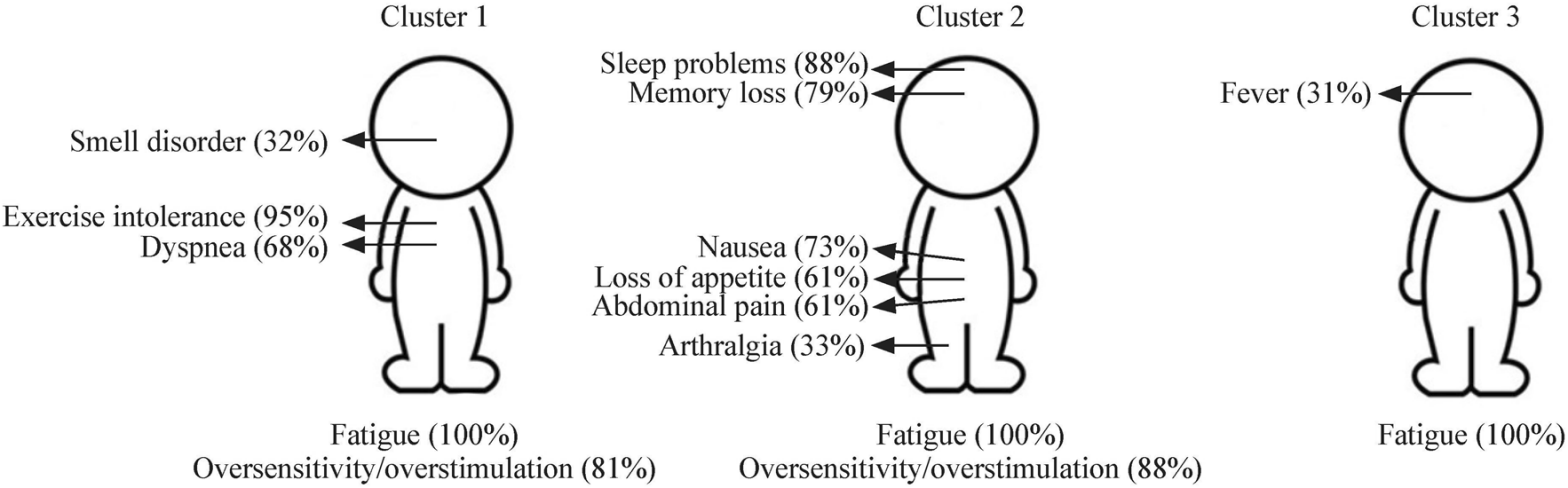
Diagram showing symptoms in each cluster. Symptoms indicated by arrows are statistically distinct between clusters and symptoms underneath are highly frequent (> 80%) within the cluster

The clusters showed differences in symptom patterns (Fig. 3). The most common symptoms are summarized in Fig. 4. Cluster 3 showed the lowest median number of symptoms per patient, with a median of five symptoms, compared to eight and ten for cluster 1 and 2 respectively (*P* < 0.001). The only symptom found more commonly in cluster 3 compared to the other clusters was fever (*P* = 0.042).

Comparing the patients’ symptoms between clusters 1 and 2 revealed two distinct symptom patterns. Symptoms that are significantly more common in cluster 1 include exercise intolerance [*P* = 0.022, odds ratio (OR) = 5.06, 1.26–26.41], smell disorder (*P* = 0.032, OR = 3.33, 1.10–12.74), and dyspnea (*P* = 0.040, OR: 2.49, 1.04–6.06). Patients in cluster 2 suffered significantly more from sleep problems (*P* < 0.001, OR = 19.48, 6.45–75.94), abdominal pain (*P* < 0.001, OR = 11.52, 4.17–35.60), nausea (*P* < 0.001, OR = 10.64, 4.06–30.45), loss of appetite (*P* = 0.001, OR = 4.32, 1.77–11.01), arthralgia (*P* = 0.024, OR: 3.31, 1.17–9.79), and memory loss (*P* = 0.032, OR = 2.80, 1.09–8.00). Myalgia was also more common in this cluster but failed to reach statistical significance (*P* = 0.081, OR: 2.35).

#### Differences in terms of impact on daily life and fatigue severity

The impact of daily-life scores followed the distribution of the number of symptoms per cluster. More symptoms correlated with a higher impact on the daily life of participants. In terms of the impact on school and exercise, patients from cluster 3 indicated that they experience significantly less impact compared to the other clusters. However, this difference was not statistically significant for social interactions, and neither were any differences found between clusters 1 and 2. For the fatigue severity as determined through the PROMIS questionnaire, cluster 3 (score: 57.0 ± 8.2) showed signs of suffering less from fatigue than the other clusters, while no significant difference (*P* = 0.077) was found between clusters 1 (score: 63.7 ± 9.4) and 2 (score: 63.7 ± 12.0). Given that all patients indicated that they suffer from fatigue, all scores were significantly higher than the average score in Dutch children (39.8 ± 12.4) [22].

#### Little distinction between clusters

Other demographic data or data related to acute COVID-19 severity showed less distinction between the clusters. No significant differences were found in the time between the infection and study visit, vaccination rate, and whether they were vaccinated before or after SARS-CoV-2 infection. We found no differences in the suspected SARS-CoV-2 variant or the number of times children were infected. While there is a significant difference in the level of education of patients, this is mainly related to age, with the younger cluster 3 showing a higher rate of elementary scholars. This difference was nullified when patients going to elementary school were excluded from the analysis (*P* = 0.30). Patients from cluster 2 showed a slightly higher rate of developing gastroenteritis during acute COVID-19, although this was not statistically significant (*P* = 0.056), while patients from cluster 3 showed a significantly higher rate of an acute COVID-19 presentation classified as “other”.

## Discussion

In this study, we clustered Dutch patients suffering from PPCC into clinically distinct phenotypes. We discovered three phenotypes that show significant differences in terms of sex, age, symptom patterns, and impact on daily life. Fatigue was the most commonly reported symptom for the entire cohort, underlined by the average PROMIS Fatigue score being significantly higher than the average score for healthy Dutch children [22].

We found three distinct phenotypes of PPCC in our pediatric population, which is in line with a study conducted in adult patients with post-COVID-19 condition by Kenny et al.[17] This study reported one cluster with more cardiorespiratory symptoms, one cluster with more musculoskeletal pain, and one cluster with a significantly lower number of symptoms and burden of disease. This may suggest that disease presentation and underlying pathophysiology of post-COVID-19 condition could be similar in children and adults. However, other studies performing cluster analyses in adult populations with post-COVID-19 condition show contrasting results, identifying post-COVID-19 condition phenotypes with different characteristics [18, 29–31]. This might be due to differences in data collection methods (self-reported versus validated questionnaires) or variations in the duration since infection (long-term symptoms after > 4 weeks versus after > 12 months). However, it could also highlight the heterogeneity of the disease, emphasizing the necessity of independent validation of cluster analyses.

Two previously described risk factors for post-COVID-19 condition in adults and children are female sex [13, 18, 31] and older age [12]. In our cohort, we found similar characteristics to be potentially associated with a higher burden of disease, as evidenced by cluster 3 mainly consisting of younger boys who experienced the least symptoms and reported the lowest impact on daily life. Another previously described predictor for PPCC is severe acute COVID-19 with or without hospitalization during the acute phase [12, 32], but because our cohort only included children with mild acute clinical presentation of COVID-19, we could not investigate this risk factor in our study.

Post-COVID symptoms had a mild to severe self-reported impact on three domains of daily life (school, social interactions, and physical functioning) for almost all our participants. This is in line with findings in a cohort of Hungarian children with PPCC [33]. However, social restrictions and lockdowns during the pandemic can also affect mental and physical health [34] and must be taken into consideration when assessing the impact of PPCC symptoms on daily life.

PPCC is a heterogeneous disease, for which the identification of three distinct clinical phenotypes may advance our understanding of the underlying pathophysiological mechanisms of the disease. One hypothesis could be that autonomic dysfunction or viral reservoirs found in the brain might play a central role in cluster 1, characterized by symptoms such as dyspnea, exercise intolerance, and neurocognitive problems [15, 35, 36]. Another hypothesis for cardiorespiratory symptoms such as dyspnea and exercise intolerance could be persistent lung inflammation, as was recently described in an adult population with PCC [37]. Cluster 2 reported high numbers of gastrointestinal complaints such as abdominal pain, nausea, and loss of appetite, which could be a result of gut microbial dysbiosis, viral persistence, or altered neuro-immune interactions in the gut [15, 38]. In cluster 3, (recurrent) fever was a common symptom, which could be a result of autoimmunity, immune dysregulation due to chronic inflammation, or dysautonomia [15, 39]. Nevertheless, this cluster had a small sample size of only 16 patients, making it challenging to draw generalizable conclusions. All described theories need further in-depth investigations, preferably through (randomized) trials.

Our study has limitations. First, we only invited children who were referred to our tertiary care clinic to participate in our study. This means we only included patients with severe PPCC symptoms, which has created a selection bias, lowering the generalizability of our results. In addition, because this was a real-time study, there was no standard timeframe after infection that children were seen for the study visit, which explains the large variability in days since infection. Furthermore, because the POCOS study included children who were referred for standard care reasons, not all data was collected for every patient, which explains why there is missing data for some participants (e.g., the PROMIS Fatigue questionnaires). Second, the selection of the number of clusters was based on visual inspection of the clustering dendrogram instead of statistical methods. For the individual imputed datasets, the Dunn index [26] suggested a wide range for the number of clusters but selected three clusters most often. Finally, our cohort consisted of 111 patients, which is a relatively small sample size to perform cluster analyses on, hence why we were unable to perform validation analyses within this study cohort.

On the other hand, we were able to perform an unbiased hierarchical clustering analysis on a population with physician-diagnosed PPCC with mild acute SARS-CoV-2 infection, where alternative diagnoses were excluded. Another strength of our study lies in the scope of information we collected, providing a complete view of our participants, while biologic samples collected in the POCOS study allow for molecular characterization of the clusters in future analyses.

Classification of PPCC phenotypes can aid in comprehending its progression, identifying its causes, and ultimately developing management strategies tailored to specific phenotypes. In addition, the identification of phenotypes can help determine appropriate, personalized rehabilitation treatment strategies for children with PPCC.

Validation of these cluster analyses in a larger population is recommended to increase generalizability. Machine learning-based clustering has previously been applied to identify potential PPCC patients based on their clinical records [40], and has also been employed in the identification of PCC phenotypes in adults [30]. However, this method has not yet been performed for PPCC cluster analyses, making it a potentially promising tool. Further biomedical research, e.g., with a multi-omics approach, is needed to determine the possible underlying pathophysiology associated with these phenotypes.

In conclusion, PPCC is a heterogeneous and poorly characterized illness that can significantly affect the lives of children. This study found three distinct clinical phenotypes of PPCC that show differences in terms of gender, age, symptom patterns, and impact on daily life. These phenotypes may reflect different underlying pathophysiological mechanisms for post-COVID symptoms, which could help categorize patients for more successful monitoring and treatment strategies, as well as funnel future research into potential cluster targets.

### Supplementary Information

Below is the link to the electronic supplementary material.

Supplementary file1 (DOCX 349 KB)

## Data Availability

The datasets generated during and/or analyzed during the current study are available from the corresponding author on reasonable request**.**
