# Supplementary material for: Clinical-based phenotypes in children with pediatric post-COVID-19 condition
Source: World J Pediatr. 2024 Apr 25;20(7):682–91. doi: 10.1007/s12519-024-00805-2 (PMC11269322; doi:10.1007/s12519-024-00805-2)
Supplement: Supplementary file 1 — Supplementary file1 (DOCX 349 KB) [file 12519_2024_805_MOESM1_ESM.docx]

**Supplementary information**

*Table S1: All variables used as data for the clustering algorithm.*

| **Category** | **Variables** |
| --- | --- |
| Demographics | Sex, Age, Body Mass Index, Level of Education |
| Comorbidities | Asthma, Allergic, Psyche |
| Complaints | Fatigue, Exercise intolerance, Memory loss, Oversensitivity/Overstimulation, Smell disorder, Taste disorder, Headache, Dizziness, Abdominal pain, Nausea, Loss of appetite, Throat pain, Fever, Arthralgia, General pain, Heart palpitations, Skin rash, Sleep problems, Concentration loss, Hair loss, Menstrual problems |
| Impact on daily life | School, Social contacts, Exercise |
| Other | Number of SARS-CoV-2 infections |

**
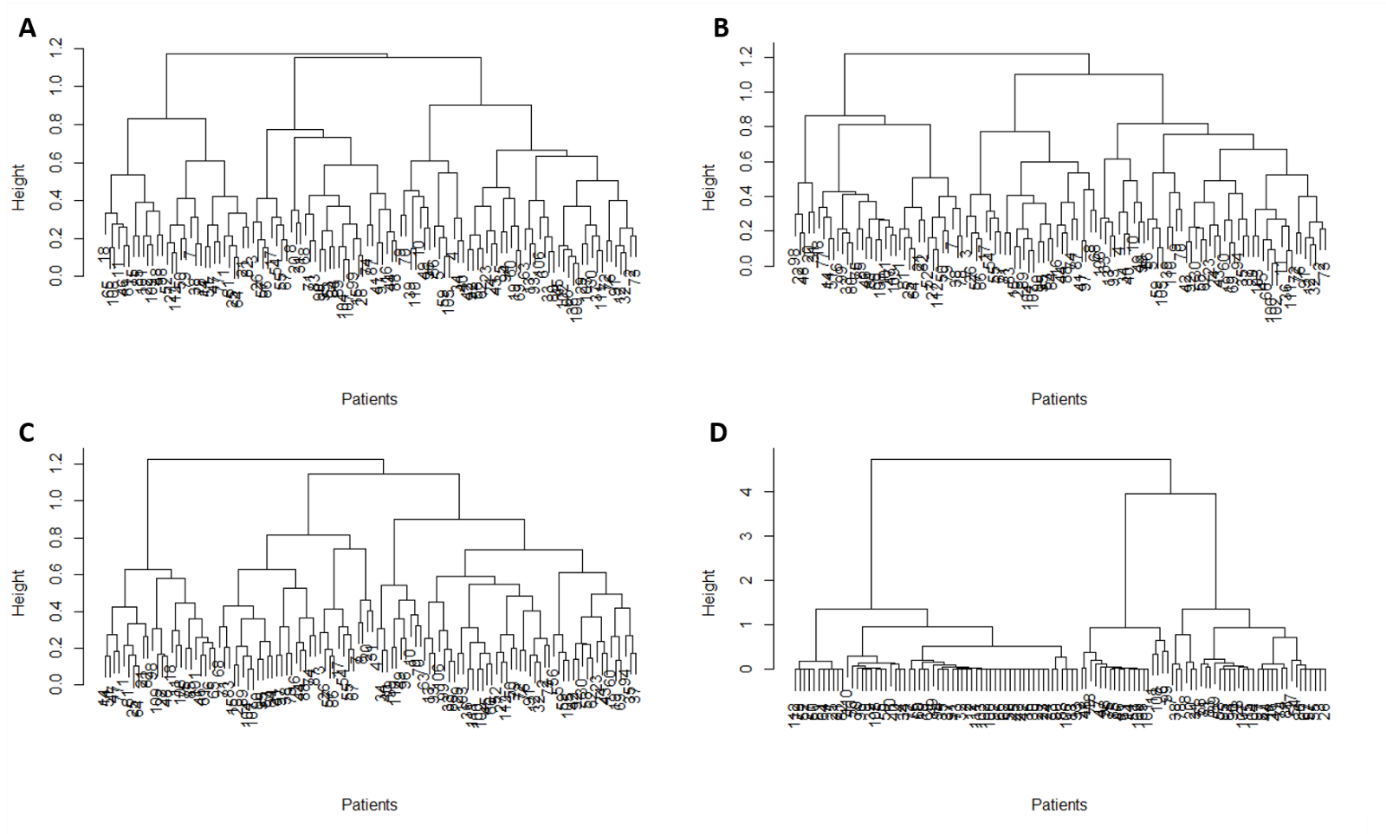
**
*Figure S1: Dendrograms from clustering efforts in 3 individual imputed datasets (A-C) and from the consensus clustering (D).*
